# Healing of colonic anastomosis in rats under obstructive ileus conditions

**DOI:** 10.15190/d.2021.8

**Published:** 2020-06-30

**Authors:** Kalliopi Despoudi, Ioannis Mantzoros, Orestis Ioannidis, Lydia Loutzidou, Panagiotis Christidis, Christos Chatzakis, Grigorios Gkasdaris, Dimitrios Raptis, Manousos George Pramateftakis, Stamatios Angelopoulos, Thomas Zaraboukas, George Koliakos, Konstantinos Tsalis

**Affiliations:** ^1^4th Academic Department of Surgery, School of Medicine, Faculty of Health Sciences, Aristotle University of Thessaloniki, Greece; ^2^Department of Pathology, School of Medicine, Faculty of Health Sciences, Aristotle University of Thessaloniki, Greece; ^3^Department of Biochemistry, School of Medicine, Aristotle University of Thessaloniki, Greece

**Keywords:** Anastomosis, ileus, leak, bursting pressure, collagen.

## Abstract

Background: The anastomosis leak in colon resections is a crucial post-operative complication with significant morbidity and mortality. Methods: Forty (40) Wistar rats were allocated in two groups. In SHAM group only anastomosis was performed. In ILEUS group anastomosis was performed following one day of ileus. Animals in both groups were subdivided in two groups according to the day they were sacrificed, 4th or 8th post-operative day. A number of variables between the groups were estimated. Results: Body weight loss was higher following obstructive ileus on both days. Adhesion score in 4th and 8th post-operative day was higher in ILEUS1, ILEUS2 groups compared to SHAM1, SHAM2 groups respectively (p<0.001 for both). Neovascularization decreased following obstructive ileus compared to control on the 4th day (ILEUS1 vs. SHAM1, p=0.038). Bursting pressure was lower in ILEUS2 group than SHAM2 group (p<0.001). The number of fibroblasts decreased following obstructive ileus compared to control on the 4th and 8th day (ILEUS1 vs. SHAM1, p=0.001, ILEUS2 vs SHAM2, p=0.016). Hydroxyproline concentration was decreased in ILEUS2 group compared to SHAM2 group (p<0.001). Conclusions: The balance of collagenolysis and collagenogenesis plays a decisive role in the healing of anastomoses following bowel obstruction. Under those circumstances, anastomosis’ bursting pressure is reduced owning to decreased neovascularization, reduced fibroblast presence and lower hydroxyproline concertation. In our study, local inflammation, neocollagen concentration and collagenase activity were not associated with this adverse effect. However, further research should delineate the mechanisms of healing of colonic anastomoses and identify those factors that can improve our outcomes.

## INTRODUCTION

Colonic surgery is attracting increasing interest due to its particular anatomical and pathophysiological characteristics and the increased incidence of colonic malignancy. Colorectal cancer is the third most common cancer in the world, and surgery is the mainstay of treatment. It is estimated that 1 out of 20 people over 75 will develop colon cancer^1,2^. A prerequisite for the success of surgery and the reduction of post-operative complications is the creation of safe anastomosis following surgical resection^3,4^. The healing of anastomoses represents a complex process of repairing tissue damage, the end result of which depends on several local and systemic factors. The healing process is the subject of many clinical and experimental studies, as it is an important prognostic factor of postoperative morbidity and mortality in colorectal cancer interventions^5-7^.

Anastomosis leakage in colon resections, especially for cancer, remains a very serious post-operative complication with significant morbidity and mortality. Despite advances in surgical techniques, suture materials, adequate preoperative preparation, and perioperative chemoprophylaxis, the rate of leakage from anastomosis has remained high (3-30%) over the last few decades^4,8-10^. In particular, it ranges from more than 17% in selective to 30% in urgent interventions. It is also known that more than 30% of patients with colorectal cancer first present with obstructive ileus symptomatology and 10% of them need urgent surgical treatment, which is accompanied by an increased incidence of anastomosis leakage^11-13^.

The healing of anastomosis after colon resection following obstructive ileus is difficult. On the one hand, the large proximal dilation of the intestine causes wall ischemia, on the other hand, the large difference in the diameters of the two parts that are anastomosed leads to an increased likelihood of anastomosis dehiscence. Additionally, ischemia of the intestinal wall, hypovolemia and electrolyte disturbances can inhibit anastomotic healing. The creation of protective ileostomy or colostomy proximal of the anastomosis, although it does not appear to affect healing, seems to reduce postoperative morbidity and mortality by protecting against possible anastomotic dehiscence^14^.

The aim of the current experimental study was to evaluate the healing of colonic anastomosis following large bowel obstruction. Anastomosis healing was evaluated by measuring anastomosis ruptures and the gravity of adhesions by measuring the bursting pressure of intact anastomoses, quantifying hydroxyproline and collagenase and evaluating histological parameters of the healing process in the area of anastomosis.

## MATERIALS AND METHODS

### Experimental animals

An experimental animal protocol was designed to minimize discomfort and pain to the animals. For this research, forty male rats of the Wistar species were used, each weighing 200-300 g. The Ethical Committee of the Department of Veterinary Services of the Prefecture of Thessaloniki approved this research (S.N.: 13/11872/11-09-08) and all necessary approved protocols for laboratory animal care were applied. The rats were under a 12-hour cycle of light and dark conditions for seven to ten days prior to the operation. Each animal was individually housed and had unlimited access to both food and water before and after the operation. No antibiotics were administrated. The sacrifice was performed using intracardial administration of KCL solution of 10% concentration.

#### Experimental groups

The animals were divided randomly into 2 groups of 20 rats each.

ILEUS GROUP (n = 20 animals): Anastomosis was performed following one day of obstructive ileus:

ILEUS1 (10 animals): the sacrifice took place on the 4^th^ post-op day

ILEUS2 (10 animals): the sacrifice took place on the 8^th^ post-op day

SHAM GROUP (n = 20 animals): Only anastomosis was performed. *SHAM1* (10 animals): the sacrifice took place on the 4^th^ post-op day *SHAM2* (10 animals): the sacrifice took place on the 8^th^ post-op day

SHAM1 (10 animals): the sacrifice took place on the 4^th^ post-op day

SHAM2 (10 animals): the sacrifice took place on the 8^th^ post-op day

### Anaesthesia and operative technique

The surgery was initiated using a 3cm incision at the midline. The anaesthesia was administered intraperitoneally using thiopental at a 40mg per kilogram concentration. A 1cm part of the colon was resected at a distance of 5cm from the rectum followed by an end-to-end anastomosis. Eight polypropylene sutures were used for the anastomosis, with 6-0 at a single layer. The obstructive ileus was initially “created” using 3-0 silk suture at a 5cm distance from the rectum. 24 hours later, a 1cm part of the large intestine around the ligation area was excised and an end-to-end anastomosis was performed. The abdominal wall was closed using 3-0 silk sutures.

### Body Weight

All animals were weighed at the start of the experiment and before sacrifice. Measurement was done with precision scales according to a code assigned to each animal after the anaesthesia was administered.

### Macroscopic examination

On the sacrifice day, the animals were subjected to laparotomy after proper administration of anaesthesia. During the operation, the anastomotic parts of the intestine were dissected and examined macroscopically. The presence of abscess near the anastomosis or peritonitis, the presence of adhesions and anastomotic integrity were evaluated using the van der Ham scale^15^. Score 0 represented no adhesion formation, score 1 a minimum number of adhesions, score 2 moderate adhesion between the anastomosis and, for example, an intestine loop and score 3 represented severe adhesion formation plus the presence of abscess.

### Bursting pressure

Bursting pressure was evaluated *ex vivo*. A 2.5cm part of the colon containing the anastomoses was removed alongside the attached adhesions. A catheter was inserted into the distal part of the removed colon after the proximal part was closed with a silk 3-0 suture. The catheter was secured at the bursting pressure measuring device. The catheter was fixed to the bursting pressure apparatus, as described in previous research^16-18^. Through this, the lumen of the bowel was filled with normal saline solution at a 1ml/min rate. The pressure, measured in mmHg, at which a leakage of normal saline or rupture occurred was defined as the bursting pressure. The exact point of a possible leak or rupture during the measurement of bursting pressure was noted, due to the fact that in some rats that point was the site of the anastomosis and in others was at another point.

### Histological assessment

After the bursting pressure measurement, the resected part of the intestine was separated from the mesentery and the fat then cleaned with normal saline. The segment of the colon containing the anastomosis was resected, with half-cm parts on each side. This was divided into two parts in the vertical level. One part of the segmented colon was placed in a formaldehyde solution of 4% concentration for histology examination and then stained with eosin-haematoxylin stain. Under a light microscope, the anastomosis was evaluated histologically using the 0-4 Erlich-Hunt grading scale with Phillips et al.’s modification^19^. The parameters included: the infiltration of inflammatory cells, the formation of new blood vessels, fibroblast activity and the deposition of collagen^20^. These parameters were evaluated individually using a numbered scale: 0 (-) = no evidence; 1 (+) = occasional evidence; 2 (++) = light scattering; 3 (+++) = abundant evidence; and 4 (++++) = confluent fibres or cells.

### Hydroxyproline

The evaluation of collagen deposition in the anastomosis was performed by evaluating the hydroxyproline percentage. For that purpose, the second part of the resected colon with the anastomosis was weighed and kept at -20°C after being cut into two segments vertically. The hydroxyproline quantity in the tissues was measured with modified methods from other experiments^21,22^. The samples were first lyophilized, then the tissue specimens were homogenized using a polytron homogenizer in distilled water. The collagen that was soluble to acid was taken from the tissue of the specimens using incubation overnight with an acetic acid solution of 0.5mol/l at 4°C. 70 μL of standard/test sample was hydrolysed in 30 μL NaOH 10.125 mol/L for 25 min at 120℃ by autoclaving. After hydrolysation, the sample was blended with a chloramines-T reagent buffer (0.056mol/L) at room temperature. The oxidation processed lasted for 25 minutes. The chromophore growth was followed by adding Ehrlich’s reagent. The absorption was assessed at 550nm using a Biotek μQuant™ spectrophotometer. The presence of hydroxyproline in unknown tissue extracts was determined from the standard curve after absorbance values were plotted against the concentration of standard hydroxyproline. The outcomes were evaluated in μg per tissue gram^21^.

### Type Ⅰ Collagenase

The density of type I collagenase was also measured in another part of the anastomotic area using an ELISA kit (USCNLIFEm E0212r). A plate was precoated with specific antibodies for type I collagenase (polyclonal, conjugated to biotin). The control sample was then added. Avidin, which was also conjugated to hydroxyproline, was also added to the plate and hatched. The development of chromophore occurred after adding TMB substrate solution. The reaction was terminated using a sulphuric acid solution. Using spectrophotometry, the change in colour was assessed at 450nm (by Stat Fax-210™ spectrophotometer (Awareness Technology Inc.)) The density of type I collagenase in random tissue specimens was extracted from the standard curve. The results were measured in μg per gram of wet tissue^23^.

Statistical analysis

The data extracted from the experiment were summarized using statistical descriptive indices of central tendency and dispersion. Data appear as mean value +/- standard deviation as they were normally distributed after the Shapiro-Wilk test for normality. ANOVA was performed to compare all the study groups. For the post hoc pairwise comparisons the independent t-test were used with the Bonferoni correction Paired before-and-after observations on the same subjects were analyzed using the paired t-test. Percentages were compared using the Fisher’s Exact Test. The level of statistical significance was set at p value <0.05 for the comparisons between the groups. All the statistical analyses were performed using the IBM SPSS Statistics (Version 22) enhanced with the module Exact Tests.

## RESULTS

### Bodyweight

**Figure 1:**
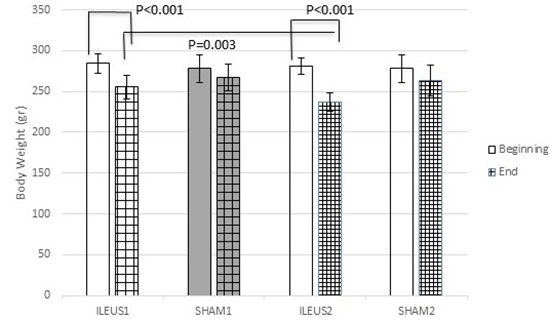
Comparative bar chart of body weight Statistically significant difference in body weight in both 4^th^ and 8^th^ post-operative day was noted in ILEUS group. In addition, body weight in ILEUS2 subgroup was statistically significant lower compared to ILEUS1 subgroup after the intervention.

The mean body weight was calculated both preoperatively and on the day of sacrifice (4^th^ and 8^th^ postoperative days). The difference between the mean values was evaluated. According to the statistical analysis, the mean weight between the subgroups did not present a statistically significant difference at the beginning of the experiment (p=0.707). However, the mean body weight differed between groups at the end of the experiment (p<0.001), as the weight in group ILEUS2 was significantly lower than in group ILEUS1 (p=0.003). Bodyweights at the beginning and end of the experiment within the same group did not differ significantly between group SHAM1 (p=0.168) and SHAM2 (p=0.09) but did differ in groups ILEUS1 (p<0.001) and ILEUS2 (p<0.001). Figure 1 presents the bodyweights at the beginning and end of the experiment.

**Figure 2:**
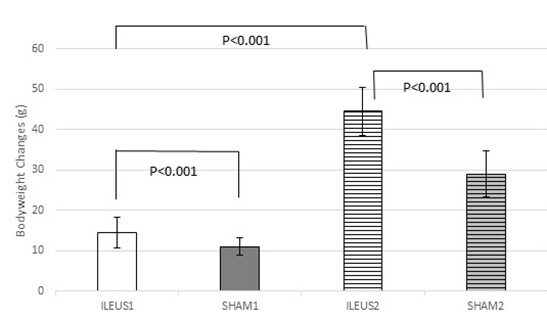
Comparative bar chart of body weight changes In ILEUS subgroup, there was a statistically significant increase in body weight changes from the fourth to the eighth day. In addition, weight changes in SHAM2 subgroup was decreased in comparison to ILEUS2 subgroup and SHAM1 in comparison to ILEUS1.

Bodyweight decreased in all experimental groups from the day of the experiment to the day of sacrifice. Bodyweight changes differed significantly among the subgroups (p < 0.001). In particular, significant differences in bodyweight change were found between subgroups SHAM1 and ILEUS1 (p < 0.001), SHAM2 and ILEUS2 (p < 0.001), and ILEUS1 and ILEUS2 (p=0.001). Figure 2 presents the bodyweight changes.

### Anastomotic dehiscence

All animals survived the experiment. Sacrifice occurred at the 4^th^ and 8^th^ postoperative days, according to the protocol of the experiment. The colon segment that was removed was examined macroscopically to determine whether the anastomosis was intact. The frequency of rupture of the anastomosis was evaluated on both the 4^th^ and 8^th^ postoperative days. On the 4^th^ postoperative day in the SHAM1 group, no rupture occurred, while in the ILEUS1 group there were 2 animals with anastomotic dehiscence (20%). However, there was no statistically significant difference in the rupture percentages (p=0.474). At the 8^th^ postoperative day, no anastomotic leak occurred in the SHAM2 group, while 3 animals presented with anastomotic leak in the ILEUS2 group (30%). However, there was no statistically significant difference in the dehiscence percentages (p=0.211). The burst of the anastomosis resulted in perianastomotic abscess and peritonitis. In addition, no difference in dehiscence percentages was noted between the SHAM1 and SHAM2 groups and the ILEUS1 and ILEUS2 groups (p=1).

### Adhesions

**Figure 3:**
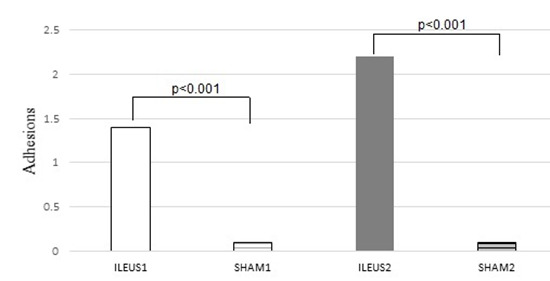
Comparative bar chart of adhesion formation (mean) The adhesion formation score was statistically significantly higher in both ILEUS subgroups compared to SHAM.

The evaluation of adhesions was done during the macroscopic examination of the anastomosis, according to the van der Hamm scale**.** According to this scale, on the 4th postoperative day, 90% of the rats showed no adhesions in subgroup SHAM1, while 10% had grade 1 adhesions. In contrast, in subgroup ILEUS1, all rats exhibited adhesions, with 80% being grade 1 and 20% grade 3. At the 8th postoperative day, 90% of the SHAM2 subgroup rats showed no adhesions, while 10% had grade 1 adhesions. In the subgroup ILEUS2, all rats showed adhesions, with 80% of them grade 2 and 20% grade 3. The adhesion formation scores differed significantly between groups (p < 0.001; Figure 3).

On both the 4^th^ and 8^th^ postoperative days, adhesion scores were significantly higher in the ILEUS1 and ILEUS2 groups compared to the SHAM1 and SHAM2 groups (p<0.001 for both). Also, while the adhesion formation score didn’t differ in the SHAM group between the 4^th^ and 8^th^ days, in the ILEUS group, adhesions increased significantly from the 4^th^ to the 8^th^ day (p=0.015).

### Bursting Pressure

**Figure 4:**
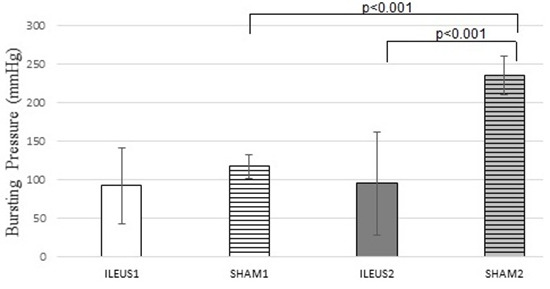
Comparative bar chart presenting bursting pressures (mmHg) (mean ± standard deviation) A statistically significant increase in the bursting pressure was noted on the eighth postoperative day in the SHAM2 subgroup compared to the ILEUS2 and SHAM1 subgroups.

The bursting pressure at the ruptured anastomosis was set at 0mm Hg. There was a significant difference in bursting pressure among groups (p < 0.001). On the 4^th^ postoperative day, the ILEUS1 group presented lower mean bursting pressure values compared to SHAM1, but the difference was not significant (p=1). However, on the 8^th^ day, the bursting pressure in the ILEUS2 group was significantly lower than in SHAM2 (p<0.001; Figure 4). Comparing the bursting pressures on the 4^th^ and 8^th^ days within each group revealed that it increased for both groups, but the difference was significant only in the SHAM group (p<0.001), and not for the ILEUS group (p=0.202).

### Leakage Site

During the evaluation of bursting pressure, the examined parts of the colon ruptured at the anastomotic site or elsewhere. On the 4^th^ day, the rupture rate at the anastomotic site was greater in the ILEUS1 group (90%) compared to the SHAM1 group (50%); however, the difference was not statistically significant (p=0.141). On the 8^th^ day, the results were analogous to the previous ones, with the ILEUS2 group presenting with a greater incidence of rupturing at the anastomotic site (80%) compared with the SHAM2 group (0%), but the difference was statistically significant (p=0.005). Comparing the leakage site on the 4^th^ and 8^th^ days within each group, it was observed that while leakage at the anastomotic site decreased from the 4^th^ to the 8^th^ day in both groups, this difference was significant for the SHAM group (p=0.033), but not for the ILEUS group (p=1).

### Histological Examination

The segments from the anastomotic region were assessed microscopically after haematoxylin and eosin staining. The classification was semiquantitative/ categorical and was based on the Erlich-Hunt scale, as amended by Philips^19^. The analysis was done in a blind fashion by an experienced pathologist. In each group, the evaluated parameters were leukocytosis, neovascularization, fibroblasts and neo-collagen generation.

### Leukocytosis

**Figure 5:**
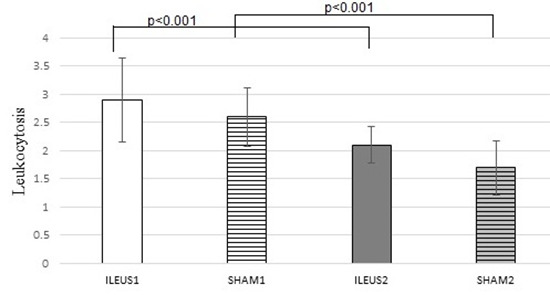
Comparative bar chart presenting the average inflammatory cell infiltration, according to the scale of Ehrlich and Hunt, as modified by Philips et al. (mean ± standard deviation) Increased leucocytosis in ILEUS1 subgroup compared to ILEUS2 subgroup was noted. In addition, increased leucocytosis was noted in SHAM1 group compared to SHAM2 group.

Statistical analysis didn’t reveal any significant differences among groups (p=0.999). Leukocytosis increased following obstructive ileus, compared to the control group, both on the 4^th^ and 8^th^ postoperative days, but none of the differences were significant. Comparing leukocytosis on the 4^th^ and 8^th^ days within each group revealed that a significant decrease from the 4^th^ to the 8^th^ day occurred in both groups (ILEUS1 vs. ILEUS2 with p < 0.001 and SHAM1 vs. SHAM2 with p < 0.001; Figure 5).

### Neovascularization

**Figure 6:**
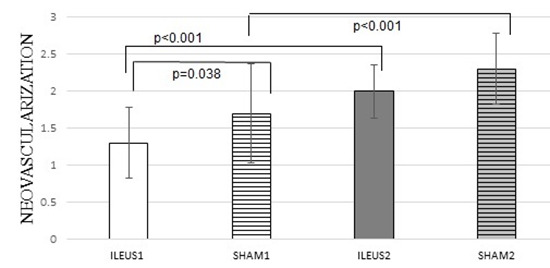
Comparative bar chart presenting the average new vessel formation (neoangiogenesis), according to the scale of Ehrlich and Hunt, as modified by Philips et al. (0–4) (mean ± standard deviation) The average neoangiogenesis was statistically significantly increased in ILEUS2 subgroup compared to ILEUS1 subgroup. In addition, increased neovascularization was noted in SHAM2 group compared to SHAM1 group. Furthermore, augmented neoangiogenesis was revealed in SHAM1 group compared to ILEUS1 group.

 Statistical analysis revealed significant differences among groups (p < 0.001). Neovascularization significantly decreased following obstructive ileus, compared to the control, on the 4^th^ day (ILEUS1 vs. SHAM1 with p=0.038), but not on the 8^th^ day (ILEUS2 vs. SHAM2 with p=0.559). Comparing neovascularization on the 4^th^ and 8^th^ days within each group revealed that a significant increase from the 4^th^ to the 8^th^ day occurred in both groups (ILEUS1 vs. ILEUS2 with p<0.001 and SHAM1 vs. SHAM2 with p<0.001; Figure 6).

### Fibroblasts

**Figure 7:**
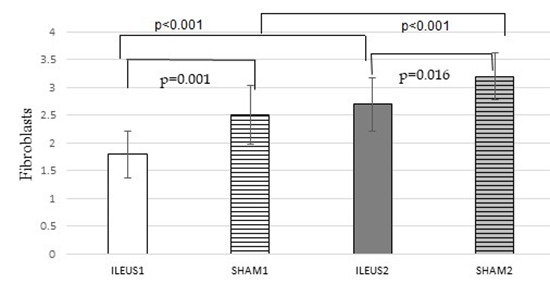
Comparative bar chart presenting the average fibroblast activity, according to the scale of Ehrlich and Hunt, as modified by Philips et al. (0–4) (mean ± standard deviation) The average fibroblast activity was statistically significantly higher in ILEUS2 subgroup compared to ILEUS1 subgroup. In addition, increased fibroblast activity was noted in SHAM2 group compared to SHAM1 group. Furthermore, augmented fibroblast activity was revealed in SHAM1 group compared to ILEUS1 group SHAM2 group compared to ILEUS2 group.

Statistical analysis revealed significant differences among groups (p < 0.001). Fibroblasts significantly decreased following obstructive ileus, compared to the control, both on the 4^th^ and on the 8^th^ days (ILEUS1 vs. SHAM1 with p=0.001 and ILEUS2 vs. SHAM2 with p=0.016). Comparing fibroblasts on the 4^th^ and 8^th^ days within each group revealed that a significant increase from the 4^th^ to the 8^th^ day occurred in both groups (ILEUS1 vs. ILEUS2 with p < 0.001 and SHAM1 vs. SHAM2 with p=0.001; Figure 7).

### Neocollagen

**Figure 8:**
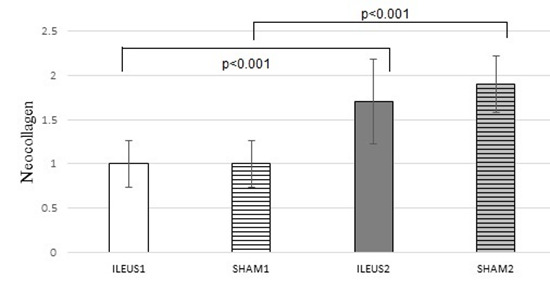
Comparative bar chart presenting the average collagen deposition, according to the scale of Ehrlich and Hunt, as modified by Philips et al. (0–4) (mean ± standard deviation) The average collagen deposition was statistically significantly higher in each subgroup in collagen deposition from the fourth to the eighth day.

Statistical analysis revealed significant differences among groups (p < 0.001). Neocollagen was not affected by obstructive ileus on the 4^th^ day and slightly decreased, but not significantly, on the 8^th^ day compared to the control (p=0.287). Comparing neocollagen on the 4^th^ and 8^th^ days within each group showed that a significant increase from the 4^th^ to the 8^th^ day occurred in both groups (ILEUS1 vs. ILEUS2 with p<0.001 and SHAM1 vs. SHAM2 with p<0.001; Figure 8).

### Hydroxyproline

**Figure 9:**
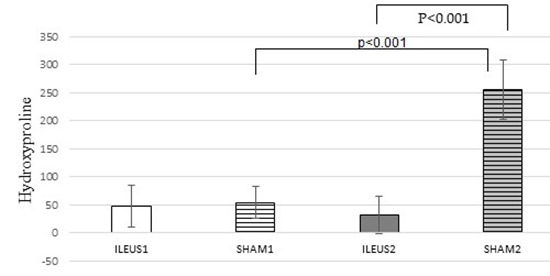
Comparative bar chart of hydroxyproline tissue contents (μg/g tissue) at the anastomotic site (mean ± standard deviation) In SHAM subgroup, there was a statistically significant increase in hydroxyproline tissue content from the fourth to the eighth day. In addition, hydroxyproline tissue contents at the anastomotic site in SHAM2 subgroup was increased in comparison to ILEUS2 subgroup.

Hydroxyproline concentration differed significantly between groups (p<0.001). Specifically, it was similar on the 4^th^ day in the ILEUS1 and SHAM1 groups (p=1), but significantly decreased on the 8^th^ day following obstructive ileus, compared to the control (ILEUS2 vs. SHAM2 with p<0.001; Figure 9). Comparing hydroxyproline on the 4^th^ and 8^th^ days within each group showed that a significant increase from the 4^th^ to the 8^th^ day occurred in the control group, but not after obstructive ileus (SHAM1 vs. SHAM2 with p<0.001 and ILEUS1 vs. ILEUS2 with p=0.664).

### Type I Collagenase

**Figure 10:**
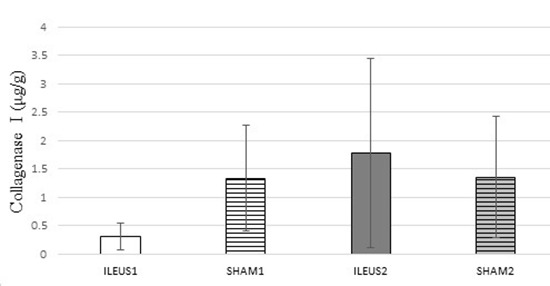
Comparative bar chart of collagenase I tissue contents (μg/g tissue) at the anastomotic site (mean ± Standard deviation) No statistically significant difference was noted.

Collagenase concentration did not differ significantly between groups (p=0.221). Specifically, while it decreased on the 4th day in the ILEUS1 group, compared to the SHAM1 group, the difference was not significant (p=0.928);

furthermore, it increased on the 8th day following obstructive ileus, compared to the control, but the difference was not significant (p=0.1). Comparing collagenase on the 4th and 8th days within each group revealed that while an increase from the 4th to the 8th day occurred in both the control group and after obstructive ileus, no significant difference was observed (Figure 10).

## DISCUSSION^8,9,24-109^

The healing of anastomosis is a complex biological procedure of restoring tissue damage. The final result depends on both regional and systemic factors. Insufficiency of the mechanisms of the anastomosis healing process results in anastomotic dehiscence, which increases postoperative morbidity and mortality^8,9^. This risk is particularly high in the case of emergency surgical treatment of obstructive colorectal ileus due to neoplasm, during which the section of the intestine carrying the neoplasm is excised, followed by primary anastomosis^24,25^.

It is well known that a high proportion of colorectal cancer (8-30%) initially manifests with obstructive ileus symptomatology^26,27^. The ileus causes disturbances in the intestinal wall perfusion above the obstruction, resulting in poor blood supply to the anastomosis and increased risk of leaking^28,29^.The prognosis of patients undergoing urgent surgery is worse than those undergoing elective surgery^30-32^. It is generally believed that excision and anastomosis at one time in the occluded colon is feasible for most patients, except those who are hemodynamically unstable or present with peritonitis^24,30^. Recent animal studies on minimizing the risk of anastomotic leak provided favourable results. Omiganan, an anti-microbial peptide, enhanced the safety of gastrointestinal anastomoses in rats with intraperitoneal sepsis^108^, while selective inhibition of hydroxylases proved beneficial^105^. However, this increases the likelihood of anastomotic dehiscence and, consequently, the development of intra-abdominal abscesses and wound infection^33,34^.

Despite the improvement in surgical techniques, colorectal surgery is accompanied by significant post-operative morbidity and mortality. The presence of anastomotic leakage or rupture is the most common complication and the most important cause of post-operative morbidity and mortality after colectomy. A rupture is defined as the complete or partial separation of the anastomosis labia, while a leak refers to the presence of intraluminal intestinal contents in the extraluminal area^33^. The frequency of leakage varies from 0.2 to 4% for colon anastomoses and from 3 to 21% for rectal anastomoses^33^. This postoperative complication leads to prolonged hospital stays, increased hospitalization costs and increased mortality (from 2.7% to 10-16%)^35^.

Escape from anastomosis is a multifactorial process that involves local and / or general factors, as mentioned above. All these factors have a common feature: they affect the composition of the new or the degradation of the old collagen, which is a key point in the healing process. In the early phase of healing, anastomosis has increased destruction of old collagen fibres and a reduced composition of new ones. This period is very important because the integrity and strength of the anastomosis depends on the complementary strength of the sutures, as the fibrin layer is very thin and cannot keep the flaps united^36^. In the first 3 to 5 days, collagen degradation is prominent 2.5cm on both sides of the anastomosis, compared to its composition. After 5-7 days, there is an increase in inflammatory response, neovascularization, fibroblasts and collagen fibres. More specifically, in the first week, the synthesis of type III collagen and fibronectin predominates, followed by the synthesis of type I collagen^37,38^. Experimental studies have shown a significant reduction in collagen concentrations in the anastomosis area during the early postoperative period^39,40^. Thus, anastomotic leakage usually occurs between 5 and 7 postoperative days (remodeling phase).

In cases of obstructive ileus, the observed pathophysiological disorders are: 1) changes in intestinal mobility, 2) intestinal content concentration prior to obstruction, 3) distension of the central part of the intestine by the addition of gases and liquids, 4) multiplication of microorganisms and bacterial translocation, 5) changes in blood flow, 6) homeostasis disturbances and 7) systematic disorders^24,41^. Excessive growth of bacteria in the occluded part of the intestine is also due to the loss of self-purification capacity. The effect of all this is the concentration of a significant amount of intestinal, fluid and air content in the part of the bowel before the obstruction^42^. That also results in increased pressure before occlusion. While normal intraluminal pressure ranges between 2 and 4 mm Hg, during occlusion it may reach up to 30 mm Hg. This increased intraluminal pressure causes both the coincidence of veins and an increase in hydrostatic pressure in the blood capillaries, with consequent increased fluid output to the intestinal lumen and to the peritoneal cavity^12,43^. Rapid growth of pathogenic microbial flora (mainly bacteroides) in the supernatant of the obstruction results in increased production of NH4, H2 and CO2. The concentration of these intestinal gasses causes further bowel dilatation^44^. Compression of the intestinal wall veins, which leads to increased diffusion of fluids from the capillaries to the intercellular space, is followed by an increase in edema of the intestinal wall and consequently the obstruction of arterioles. This results in ischemia, which is more pronounced initially in the intestinal mucosa. The prolongation of ischemia leads to necrosis, perforation of the intestine, excretion of intestinal contents in the peritoneal cavity and peritonitis^44,45^. The ileus also disrupts the intestinal barrier, resulting in the entry of microbes and endotoxins into systemic circulation via the portal vein. Within 6-12 hours of the occlusion, microorganisms are found within the peritoneal cavity (bacterial translocation), and systemic endotoxemia begins on the 4^th^ day. Endotoxemia may lead to the development of Systemic Inflammatory Reaction Syndrome (SIRS) or Multidrug Disorder Syndrome (MODS)^46,47^.

It is known that 8-30% of patients with colorectal cancer first appear with obstructive ileus symptomatology and 10% of these require urgent surgical treatment. Obstruction is usually found in the left colon, as its diameter is shorter and its content denser^29,42^. The ileus causes the reported local and systemic disorders, which inhibit the anastomosis healing process and lead to an increased frequency of dehiscence of the anastomosis. More specifically, the ileus causes the following disorders in the healing of anastomoses: disruption of anastomosis circulation and disruption of bowel wall. Mucosal haemorrhage, especially of the submucosal layer, determines perfusion of the anastomosis. It is known that the capillary network of the colon is less developed than that of the small intestine, which also explains its greater susceptibility to decreased blood flow^13,22^. In the obstructive ileus, the increased intraluminal pressure causes ischemia of the intestinal wall. In particular, the mucosa is edematous and hypo-perfused, and extensive swelling develops in the submucosal layer. Also, as the ischaemia is prolonged, ulcerations in the intestinal mucosa develop due to destruction of the epithelial cells. Ischemic lesions at this stage are usually reversible. Over the time, however, the lesions extend to the musculoskeletal and the entire thickness of the wall and are irreversible, which can lead to perforation of the intestine^41,43^. After the cause of the obstructive ileus has been removed, the ischemic bowel is reperfused, and there is a possibility of ischemia-reperfusion injuries^43,44^. This results in severe tissue alterations in the intestinal epithelium and capillary endothelium due to the release of oxygen free radicals. Ischemic lesions, destruction of the parietal capillaries and secretion of vasoconstrictor substances result in disorder of the anastomosis infusion and inhibit its healing^45,48^. Experimental studies have shown that ileus increases the activity of metalloproteinases, which reduce the deposition of new collagen in the anastomosis and increase the degradation of the old. Their greater concentration and consequent greater collagen degradation is observed near the staple line. The end result is a delay in the healing of anastomosis and an increased chance of leakage^42^. Newer studies demonstrated that selective matrix metalloproteinases (MMP)’ inhibition increased anastomotic breaking strength^106^ and the the application of protective local agents, such as albumin/glutaraldehyde, in colonic anastomoses leads to better outcomes^109^.

Patients with colorectal cancer, particularly those with an obstructive ileus condition, show a decrease in body weight^49,50^. It has also been found that subnutrition and hypoproteinaemia inhibit healing of anastomoses, as they reduce collagen synthesis^51^. In an experimental study of obstructive ileus and tacrolimus, Raptis et al.^23^ observed greater reduction in the body weights of the experimental animals in the ileus group than in the other group. In our study, both on the 4^th^ and 8^th^ postoperative days, the presence of obstructive ileus led to significant body weight loss compared to the control.

The rate of anastomotic leakage is the most important indicator for the inadequacy of the mechanisms for healing bowel anastomoses, ranging from 3 to 30% ^52-55^. In occlusive ileus, the risk of anastomotic leakage is greater^28,56^. Raptis et al.^23^ found that leaks were more common in the ileus group than in the control group. Similarly, in our study, the frequency of rupture from the anastomosis increased in obstructive ileus conditions. Specifically, on the 4^th^ postoperative day, anastomosis leakage was observed in 20% with obstructive ileus, but the difference with the control was not statistically significant. Similarly, on the 8^th^ postoperative day, anastomotic leakage presented in 30% of rats with ileus, but this difference was also not statistically significant.

Adhesions occur when the organs of the peritoneal cavity and/or the peritoneal wall are traumatized. These are fibrous bands that connect the organs of the peritoneal cavity, either with each other or with the peritoneal peritoneum, and can contribute to microleaks from the anastomosis site, while improving perfusion^56,57^. Adhesion formation is determined by the severity of intestinal trauma, the activation of fibroblasts, the action of metalloproteinases and the adequacy of the fibrinolytic mechanism^58-60^. The quantitative evaluation of adhesions in our study was done according to the van der Hamm scale in all subgroups ^61^. Hoffman et al.^57^ also reported that adhesions occur more frequently in obstructive ileus. In the current experimental study, both in the 4^th^ and 8^th^ postoperative days, the obstructive ileus subgroup showed significantly more adhesions compared to the control group.

The adequacy of colonic anastomosis healing is assessed by measuring the anastomosis bursting pressure, which is the most reliable method of mechanical power assessment. The bursting pressure is measured by the resistance of the intestinal wall to increased intraluminal pressure^62^. During the early post-operative period (between day 3 and day 5), there is a dramatic reduction in the collagen concentration of up to 40%, due to increased collagenolysis, closer to the anastomosis site. This explains why intestinal disruption in these cases occurs in the area of ​​reperfusion^59,63^. The occurrence of rupture outside of anastomosis occurs in labile areas or in focal necrosis sites. With the progressive increase in collagen synthesis from day 5, the strength of anastomosis increases, resulting in the seventh postoperative day to gain 50% of its measured potency. After the 14^th^ postoperative day, the sutures no longer contribute to the strength of the anastomosis and rupture usually occurs away from the anastomosis^39^. Measurement of anastomosis bursting pressure is the most reliable method of assessing mechanical strength in the first postoperative week and is affected by the amount of collagen deposited^37,53,64,65^. Obstructive ileus also affects the mechanical strength of anastomoses^27,65^. Raptis et al.^23^ found a decrease in mean bursting pressure in the ileus group relative to the control group. In our study, on the 4^th^ postoperative day, while the obstructive ileus subgroup showed lower mean bursting pressure compared to the control, the difference was not significant. However, on the 8^th^ postoperative day, mean bursting pressure in the ileus subgroup was statistically significantly lower compared to control subgroup.

An essential element for evaluating the effects of bursting pressure and assessing the adequacy of healing mechanisms is the point of the bowel part where a rupture occurs in the performance of the corresponding test^10,66^. Ruptures in the area of ​​the anastomosis occur in less-powerful mechanical anastomoses or anastomoses found in early healing (3^rd^ to 5^th^ day). In contrast, ruptures away from anastomosis occur in very strong mechanical anastomoses or anastomoses, where the healing process is at an advanced stage, particularly in the remodeling phase^67,68^. Raptis et al.^23^ found that the rupture rate on anastomosis was higher in the ileus group relative to the control group. In our study, on the fourth postoperative day, the highest incidence of anastomosis rupture was observed after obstructive ileus conditions compared to the control group, but this difference was not statistically significant. However, on the 8^th^ postoperative day, a statistically significantly highest incidence of anastomosis rupture was observed after obstructive ileus conditions compared to the control group.

Healing consists of chained biological reactions that lead to collagen deposition at the anastomosis^69-71^. The most important stage of the healing process is the inflammation phase, in which a fibrin clot is formed. That clot bridges the trauma gap. Inflammatory cells (neutrophils, macrophages) also accumulate in the area. Neutrophils remove foreign bodies and necrotized tissues by releasing proteolytic factors like collagenase. Also, macrophages produce growth factors, which are responsible for the migration of fibroblasts and epithelial and endothelial cells to the wound site, resulting in the formation of granulomatous tissue^69,70,72^. The extension or inhibition of this phase may cause disruption of the anastomosis healing mechanisms. Although the process of healing the anastomoses of the colon is similar to that of skin, there are several differences particular to the gastrointestinal tract^73-75^. First, the intestinal mucosal epithelium has greater regenerative capacity, leading to faster wound repair. Anastomoses of the digestive tract acquire their maximum strength in 14-21 days, while skin sutures can take as long as the 120th day^70,74,76^. During the first 3-5 days, collagenolysis is predominant in the area of ​​anastomosis. This period is the most critical, as the sheared ends of the bowel are held together only by sutures or clips. The strength of the anastomosis gradually increases until the 12th-14th days^77^. Also, smooth muscle cells and fibroblasts are responsible for the production of collagen in the intestine, as opposed to the skin, where these cells do not play such a role. Between the 3rd and 5th days, an abundance of undifferentiated mesenchymal cells accompanies the invasion of the capillaries in the muscle layer. These cells are transformed into smooth muscle cells and phagocytes^77,78^. The formation of smooth muscle cells at the wound edges is responsible for the reshaping of the lost smooth muscle tissue. Additional factors that differentiate the intestine from the skin and other tissues are its multi-layered architecture, its high content of micro-organisms, the effect of serum on the formation of a barrier on the staple line and its particular vascularization^74^.

The healing of the anastomoses of the colon involves succession and complex processes^70,76,79^. It evolves immediately after trauma for the structural and functional repair of the colon tissues. All processes involve the epithelial and fibrous connective tissue with its three components—cells, matrix and fibres—and aim to synthesize new connective tissue and heal the anastomosis^73,79-82^. The mechanisms of healing are distinguished in four overlapping phases:^80-88^ A) haemostasis phase, B) inflammatory phase, C) production phase and D) remodeling phase. Haemostasis is distinguished in the vascular phase where there is a contraction of the bleeding vessels, the platelet phase, where platelets accumulate and the platelet or temporal thrombus is formed, and the coagulation phase^89^. The inflammation phase follows the haemostasis phase, beginning 24 hours after the wound and lasting for 3-4 days. The accumulation of platelets is followed by the infiltration of the area by white blood cells, which arrive after their dissection from the vascular wall^90^. The transudation is neutrophilic polymorphonuclear, mononuclear macrophages and lymphocytes. Immune system cells significantly contribute to the regulation of the anastomosis healing process, through the secretion of molecular signals, such as cytokines, lymphokines and growth factors^91^. The activity of polymorphonuclear cells involves the phagocytosis of bacteria (by opsonization), release of collagenase and elastase and the degradation of elastin, the basal membrane of the vessels. In addition, they produce IL-1 and TNF-α, which activate fibroblasts. In the current study, while leukocytosis increased following obstructive ileus, none of the differences observed were significant compared to the control.

The proliferation phase begins after the 3^rd^ day and ends on the 12^th^ day of healing. It involves the synthesis of new collagen, angiogenesis and the replacement of temporary fibrous tissue from granular tissue. Granular tissue is a loose connective tissue of fibrin, fibronectin, collagen fibres, glycosaminoglycans and hyaluronic acid^38,92^. In addition, it contains macrophages, fibroblasts and neoplastic vessels. Its presence lasts from day 3 to day 21 and is determined by various substances produced in the inflammatory phase^37,93^. In the productive phase of healing, which begins 6-7 days after anastomosis and is completed in 12-16 days, neovascularization also plays an important role. In particular, endothelial cells from the adjacent capillaries invade the anastomosis and form tubular formations surrounded by collagen fibres and the primary capillaries. This process is regulated by the action of promoters and inhibiters. Promoter agents include FGF, TGF-α and VEGF. Turlu et al. showed that TGF-β1 represents a potential therapeutic agent for the prevention of anastomotic leakage by increasing collagen synthesis and collagen deposition^104^. Positive effects also have local factors, such as hypoxia, low pH and high levels of lactic acid^89^. It has also been found that the ileus causes disturbances in the intestinal wall perfusion. Raptis et al.^23^ found a significant decrease in neovascularization in the ileus group compared to the control group. In our study, on the 4^th^ postoperative day, the obstructive ileus group showed lower neovascularization compared to the control and this difference was statistically significant. However, on the 8^th^ postoperative day, while neovascularization was reduced in ileus subgroup, the decrease was not significant compared to the control.

During the production phase, the area of ​​anastomosis is predominantly occupied by endothelial cells and fibroblasts. The most important processes, both in this phase and in the remodeling phase, are collagenolysis and collagenogenesis, the balance of which plays a decisive role in the healing of anastomoses. A significant increase in collagenase was found in the gastrointestinal tract following anastomosis in the colon^75,94^. An important factor directly affecting collagenase activity is the presence of bacteria in the anastomosis. Bacteria are sources of proteolytic enzymes that produce collagenase and can cause increased collagen breakdown at the site of anastomosis^95,96^. Studies on mice suggested that drinking a phosphate-based polymer can achieve the goal of preventing anastomotic leak by suppressing collagenase production in E. faecalis^107^. After the early phase, the production phase of healing is followed by the production of collagen by the fibroblasts, which increases the strength of the anastomosis^39,40,92^. For this reason, the production and deposition of collagen in the area of ​​anastomosis is an important indicator of the adequacy of the healing process^52,63^. Raptis et al.^23^ found a significant reduction in the number of fibroblasts in the ileus group compared to the control group. In our study, on the 4^th^ postoperative day, a reduced number of fibroblasts, compared with the control, was observed in the ileus subgroup and this difference was statistically significant. Similarly, on the 8^th^ postoperative day in the ileus subgroup, a decreased number of fibroblasts was observed, compared to the control, and this difference was also statistically significant.

At the remodeling phase, collagenolysis progressively decreases, whereas collagen deposition, which begins on days 7-8 from the formation of anastomosis, increases and may take up to 90 days. The collagen fibres intertwine with each other and, in combination with the connective tissue, provide elasticity, durability and integrity. In the remodeling phase, the most important event is the collagen deposition and the gradual replacement of granular tissue with connective tissue, which consists of a network of collagen and elastic fibres^38^. Additionally, as the inflammatory elements recede, angiogenesis is disrupted, fibroblasts are produced, the old type III collagen degrades and the novel type I collagen is synthesized. The strength of the anastomosis is determined by the quality and amount of the newly formed collagen. This increases from the 2nd to the 6th week of healing, then stays constant and reaches its maximum in 2-3 years. At the same time, tissue remodeling (after three weeks), which lasts up to three years, can also cause anastomosis constriction after a significant period of time (up to six months). The end result is the formation of scarring connective tissue^59,83,97^. In experimental studies, occlusive ileus has been shown to increase the activity of metalloproteinases (between day 1 and day 5), which reduces the deposition of new collagen and increases the degradation of the old, both in the anastomosis region and away from it^96,98,99^. Raptis et al.^23^ found significant reduction in neocollagen formation in the ileus group compared to the control group. Also, Moran et al.^100^, in an experimental study on rats, after erythropoietin administration under ileus conditions, found that the formation of neo-collagen decreased in the ileus group compared to all other groups. However, in our study, we didn’t observe any significant differences in neocollagen synthesis among the experimental groups.

The mechanical strength of anastomosis is dependent on connective tissue cell proliferation, collagen molecule synthesis and collagen fibril formation. The synthesis and deposition of collagen are complex biological processes that are affected by a variety of factors. Experimental models are necessary to investigate the deposition of collagen during the anastomosis healing process. The most common method is to determine hydroxyproline, as a unit of total collagen count, in colorectal cells from the anastomosis site. Hydroxyproline is one of the key amino acids components of collagen of all types and is a product of proline hydroxylation metabolism. The main feature of this amino acid is that it is almost exclusively found in connective tissue collagen. The measurement of hydroxyproline is quantitative and does not give information on the quality of collagen and its identification in fractions (types I, III and IV). However, its measurement is now the only reliable quantitative method for determining collagen in the anastomosis region^101^. It was also found that obstructive ileus causes disorders in the structure of the intestinal wall, resulting in a decrease in the concentration of hydroxyproline^63,101^. Raptis et al.^23^ found that the ileus group had a decreased concentration of hydroxyproline relative to all other groups. In our study, we found that on the 4th postoperative day, after obstructive ileus, there was a decreased concentration of hydroxyproline compared to the control, but this difference was not statistically significant, while on the 8^th^ postoperative day, there was a significant reduction in hydroxyproline concertation in the ileus group compared to the control.

Collagen is an abundant extracellular substance and determines the non-mechanical stability of connective tissue during healing. The most important types are type I collagen, which is responsible for the formation of mature collagen, and type III collagen, which occurs mainly during the early phase of healing and has less mechanical strength. Their content and proportion determine the mechanical strength and stability of the anastomosis. The total degradation of collagen is the result of the synergistic action of various MMPs^46,60,66^. Various experimental studies have shown that the intense activity of MMPs is one of the major pathogenetic factors of postoperative attenuation of the anastomosis. Other studies have found that the levels of MMPs on the suturing line increased during the first three days after anastomosis. In addition, other studies have shown that MMP-1 and MMP-13 are found in all patients with leakage, suggesting a potential predisposition for collagen metabolism disorder. These findings suggest that treatment with synthetic inhibitors of MMPs contributes to maintaining mature collagen fibres in the submucosal layer and, consequently, the integrity of anastomosis^98,102^.

Experimental studies have shown that ileus increases the activity of metalloproteinases, which reduces the deposition of new collagen in the anastomosis and increases the degradation of the old one. Their greater concentration and greater collagen degradation are observed near the staple line. The end result is a delay in the healing of anastomosis and an increased chance of leakage^42,96,98^. Collagen is resistant to the action of proteolytic enzymes and is degraded by collagenase. A significant increase in collagenase was found in the gastrointestinal tract following anastomosis in the colon^96^. Collagenase plays an important role in determining the integrity of anastomosis and the ability to hold sutures in the early postoperative days of healing. In addition, immunohistochemical studies have shown that collagenase and other metalloproteinases are proximal to the anastomosis stapling line. Collagenase belonging to the metalloproteinase family is considered to be responsible for the loss of mature collagen of the submucosal layer and the consequent breakdown of anastomosis^103^. This is supported by experimental data indicating maximum collagenolytic activity in anastomosis in rats on the 3rd postoperative day. Other experimental studies in rats using aprotinin, which is a protein inhibitor, showed a significant increase in the breakdown pressure of the anastomoses of the colon^52^. Collagenase is the major breakdown enzyme of mature collagen, belongs to the family of metalloproteinases and plays an important role in the integrity of anastomosis. The factors that affect the activity of collagenase are bacteria and the inflammatory reaction around the anastomosis. It has also been found that occlusive ileum increases the enzymatic activity of metalloproteinases and causes rapid degradation of collagen in the intestinal wall. Therefore, the collagenase concentration assay provides a better measure of the balance between collagenogenesis and collagenolysis^28,46,60^. In our study, on the 4^th^ postoperative day in the ileus subgroup, the collagenase concentration decreased in comparison with the control group, but this difference was not statistically significant. On the 8^th^ postoperative day, the ileus subgroup showed a higher collagenase concentration compared to control subgroup, but this difference was also not statistically significant.

## CONCLUSION

As a high proportion of colorectal cancer cases initially manifest with obstructive ileus symptomatology, there is a need for improved outcomes and safety of colonic anastomosis. The balance of collagenolysis and collagenogenesis plays a decisive role in the healing of anastomoses. Anastomotic healing in the early period seems to be impaired following obstructing ileus conditions, as anastomotic dehiscence percentages did not significantly increase, the bursting pressure significantly decreased, and the majority of leakages occurred at the anastomotic site. This reduction was not affected by local inflammatory reaction, neocollagen concentration and collagenase activity, but rather by decreased neovascularization, reduced fibroblast presence and lower hydroxyproline concertation. Thus, extreme caution should be exercised in the construction of an anastomosis following obstructive ileus and there is a need for measures, either local or systemic, to enhance the anastomotic healing process. Future studies should focus on the delineation of the mechanisms of healing of colonic anastomoses and identify those factors that can be altered to minimize the incidence of the anastomosis rupture.

## KEY POINTS

**◊** The balance of collagenolysis and collagenogenesis plays a decisive role in the healing of anastomoses

**◊** Obstructive ileus subgroup presented with decreased number of fibroblasts (production and deposition of collagen) and decreased concentration of hydroxyproline (reliable quantitative method for determining collagen)

**◊** In obstructive ileus subgroup, higher collagenase concentration and lower neovascularization were observed

**◊** The highest incidence of anastomosis rupture was seen after obstructive ileus conditions compared to the control group

**◊** Future studies should focus on the delineation of the mechanisms of healing of colonic anastomoses and identifying those factors that can be altered to minimize the incidence of anastomosis rupture
